# How to Perform Intravesical Chemotherapy after Second TURBT for Non-Muscle-Invasive Bladder Cancer: A Single-Center Experience

**DOI:** 10.3390/jcm12010169

**Published:** 2022-12-26

**Authors:** Zhen Li, Nienie Qi, Zhimin Gao, Li Ding, Jiawei Zhu, Qingxiang Guo, Junqi Wang, Rumin Wen, Hailong Li

**Affiliations:** 1 Department of Urology, The Affiliated Hospital of Xuzhou Medical University, Xuzhou 221000, China; 2 Graduate School, Xuzhou Medical University, Xuzhou 221000, China

**Keywords:** second-look TURBT, residual tumor, urinary bladder neoplasm, chemotherapy

## Abstract

Purpose: The objective of this study aimed to explore whether the original IVC regimen should be continued after the second TURBT or whether the IVC induction phase should be restarted from the beginning. Methods: A retrospective analysis was performed on 137 patients who underwent a second TURBT at the Affiliated Hospital of Xuzhou Medical University between April 2014 and June 2022. Based on the pathological findings, patients were divided into two groups: group A patients, who did not have a residual tumor on pathological examination after the second TURBT; and group B patients, who had residual tumor. Recurrence was determined using cystoscopy and imaging every three months. The endpoint was recurrence-free survival. Result: In the entire cohort, there was a statistically significant difference in the RFS between patients in the two IVC regimens (*p* = 0.029). The RFS of patients in group B1 was significantly lower than that of patients in group B2 (*p* = 0.009). There was no significant difference in RFS between the subgroups A1 and A2 (*p* = 0.560). Multivariate Cox regression analysis confirmed that the IVC regimen after a second TURBT (*p* = 0.012) and T stage after a second TURBT (*p* = 0.005) were both independent predictors for patient RFS. Conclusion: If the pathological findings of the second TURBT specimen is benign, patients can continue their previous treatment regimen without restarting an IVC induction phase. Unnecessary IVC can be avoided in these patients. In contrast, for patients with residual tumors in the second TURBT specimen, the need to restart the IVC induction phase should be emphasized to improve patient prognosis.

## 1. Introduction

Bladder cancer (BC) is one of the most common cancers in the world. Most patients with BC present with the disease confined to the mucosa (carcinoma in situ [CIS] and Ta stage) or submucosa (T1 stage). Non-muscle invasive bladder cancer (NMIBC) accounts for 70% of BC [1,2]. A transurethral resection of the bladder tumor (TURBT) is the gold standard treatment for patients with NMIBC [3,4]. However, many patients with NMIBC experience recurrence or progression to muscle-invasive bladder cancer (MIBC). Therefore, all patients with BC should be considered for postoperative intravesical chemotherapy (IVC). Patients may experience recurrence even after post-resection IVC [5]. Residual lesions have been reported in approximately 40% of patients with high-grade Ta and up to 55% of patients with T1 tumors after the initial resection [6,7]. Most residual lesions are detected in the original tumor bed [8]. The prognosis of patients with NMIBC can be improved with a second TURBT [9]. Therefore, many guidelines recommend that patients with high-grade Ta and/or T1 tumors should undergo a second TURBT [10].

The European Association of Urology (EAU) guidelines recommend immediate single instillation (SI) after TURBT, with no exception for the second TURBT. Meanwhile, both the American Urological Association (AUA) and EAU guidelines recommend further adjuvant IVC after TURBT [11,12]. However, there is no consensus regarding the optimal IVC regimen after the second TURBT. This study aimed to explore whether the original IVC regimen should be continued after the second TURBT or the IVC induction phase should be restarted from the beginning. No IVC protocol after the second TURBT has been clearly reported in the literature, and we are the first medical center to initiate this research.

## 2. Materials and Methods

### 2.1. Patients 

A retrospective analysis was performed on 137 patients who underwent a second TURBT at the Affiliated Hospital of Xuzhou Medical University between April 2014 and June 2022. The inclusion criteria were as follows: (1) first TURBT pathology was clearly uroepithelial carcinoma of the bladder, and NMIBC with Tis, Ta, or T1 stage was diagnosed on pathology at the initial resection; (2) patients who visited our hospital for a second resection within 2-6 weeks after the primary surgery; (3) immediate bladder perfusion therapy was performed within 24 hours after the primary resection, and thereafter, once a week; (4) the risk groups of patients were intermediate-risk or high-risk. The exclusion criteria were as follows: (1) patient data were missing or incomplete; (2) the patient’s initial resection was not performed at our hospital; (3) patient refused IVC or Bacillus Calmette-Guérin (BCG) was perfused after the primary resection; (4) underlying co-morbidities. During the second TURBT, all visible tumors and scars from the previous surgery were resected. A single chemotherapeutic drug was used for IVC in each patient. Based on the pathological findings, patients were divided into two groups: group A patients, who did not have a residual tumor on pathological examination after the second TURBT; and group B patients, who had residual tumor. The patients in both groups received two different IVC regimens. Individual regimens are carefully described in the treatment schedule. After the second TURBT, recurrence was determined using cystoscopy and imaging every 3 months. The endpoint was recurrence-free survival (RFS). RFS was defined as the duration from the date of a second TURBT to relapse, progression, or post-surgical death. 

### 2.2. Treatment Schedule

The main drugs used for IVC after TURBT at our medical center are epirubicin and pirarubicin. Although BCG is more effective, it is rarely used at our center due to its high cost in China. Epirubicin or pirarubicin (40 mg) was dissolved in 40 ml of normal saline, which was subsequently instilled into an empty bladder through a sterile catheter. Patients were instructed to refrain from voiding for 2 hours after instillation. All patients received an SI immediately after the initial TURBT. The IVC schedule after the initial TURBT was divided into induction phase (once a week for 8 weeks starting from the first week after the TURBT) and maintenance phase (once a month for 10 months). The patients underwent a second TURBT 2-6 weeks after the initial TURBT. All patients received SI of the chemotherapeutic drugs immediately after the second TURBT, and had two options. Patients were continued on the IVC cycle after the initial TURBT (original IVC regimen) or the induction phase was restarted (new round of IVC).

### 2.3. Pathology

Pathological staging of the specimens was performed at the local pathology department using the International Union Against Cancer (UICC) TNM staging of bladder cancer (2017, 8th edition) and the 2004 WHO bladder cancer grading system. All pathological specimens were re-evaluated by a dedicated bladder cancer pathologist, especially for specimens obtained prior to 2017.

### 2.4. Clinical and Pathological Characteristics

Clinicopathological data were collected, including sex, age, body mass index (BMI), smoking and gross hematuria history, stage, grade, number, and maximum diameter of the tumor, and IVC regimen after the second TURBT.

### 2.5. Statistical Analysis

All the data were transformed into categorical variables and expressed as numbers and percentages. The chi-square test was used to compare variables between the groups. Kaplan–Meier survival curves were compared using the log-rank test. Univariate and multivariate Cox regression analyses were performed to identify the independent risk factors associated with patient survival. The results were reported as hazard ratios (HR) with 95% confidence intervals (CIs). All analyses were performed using SPSS 26.0 (IBM Corp., New York, NY, USA) and R 4.1.2 (https://www.R-project.org, accessed on 22 September 2022). All statistical analyses were two-sided, and statistical significance was set at *p* < 0.05.

## 3. Results 

### 3.1. Clinical and Pathological Characteristics

**Figure 1:**
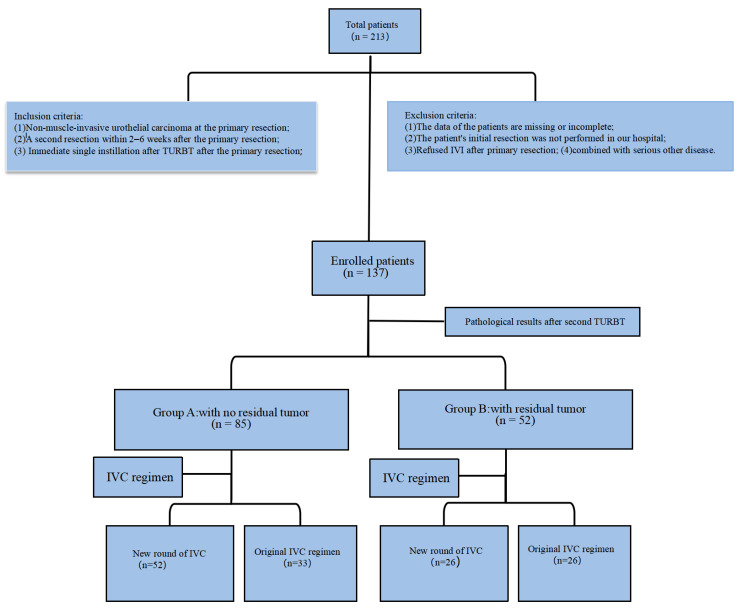
Flow chart of allocation.

Between April 2014 and June 2022, 137 patients were enrolled in this study which was conducted at our medical center (Figure 1). Group A included 85 (62%) patients who presented with benign tissue for the second TURBT, while Group B included 52 (38%) patients who presented with a residual tumor. No significant differences in BMI, age, sex, or gross hematuria history were observed between the groups. The pathological findings of most patients in Group B after the second TURBT were stage T1 (60%) and high-grade (85%) tumors. In group A, 61% of the patients (group A1) restarted the IVC induction phase, and 39% (group A2) continued with the original regimen. In group B, 50% of the patients (group B1) restarted the IVC induction phase, and 50% (group B2) continued with the original regimen (Table 1).

### 3.2. Kaplan–Meier Survival Analysis

**Figure 2:**
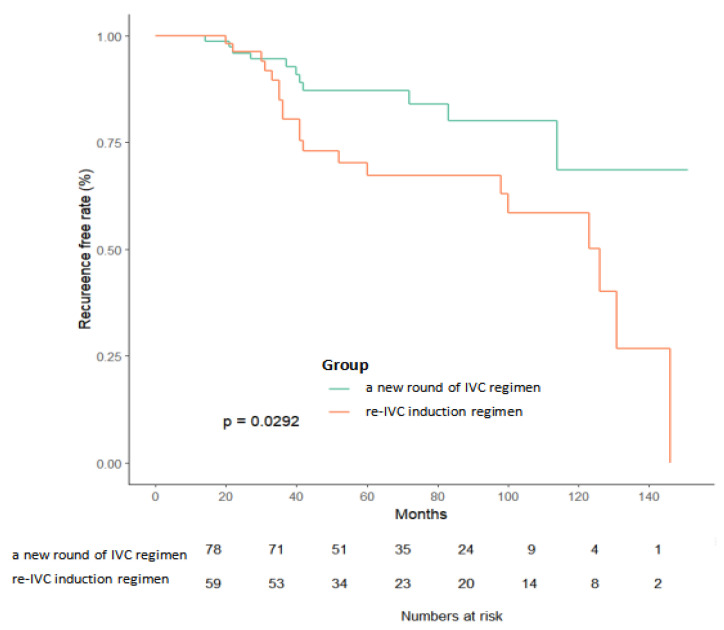
Non-recurrence curve after TURBT in two groups. Non-recurrence rate of the new round group was significantly higher than that of the original group (*p* = 0.0292) in total study cohort.

**Figure 3:**
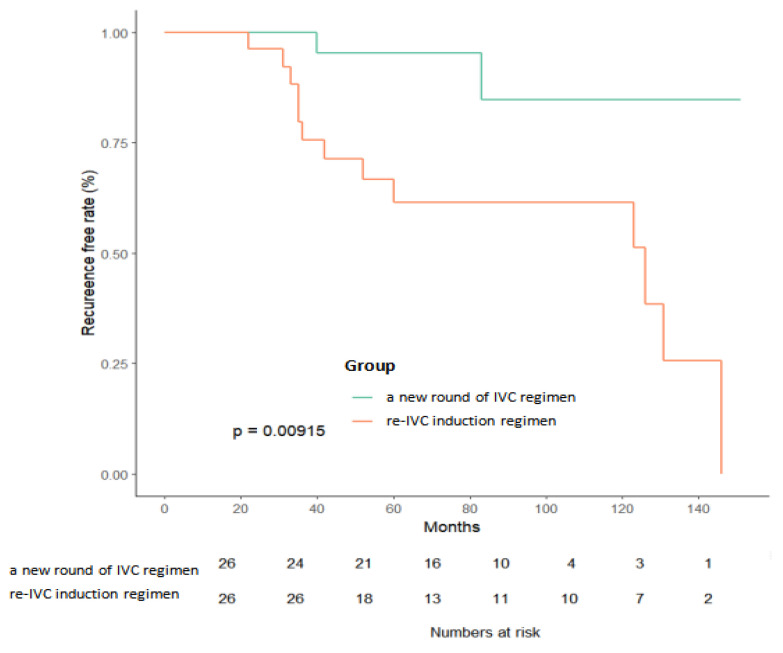
Non-recurrence curve after TURBT in two groups. Non-recurrence rate of the new round group was significantly higher than that of the original group (*p* = 0.0292) in patients who presented with a residual tumor.

The median follow-up periods were 41 and 67 months in groups A and B, respectively. Approximately 31% and 29% of patients in groups A1 and A2, respectively, experienced tumor recurrence. The recurrence rates in group B were 29% (group B1) and 44% (group B2). In the entire cohort, there was a statistically significant difference in the RFS between patients in the two IVC regimens (*p* = 0.029) (Figure 2). The RFS of patients in group B1 was significantly lower than that of patients in group B2 (*p* = 0.009) (Figure 3). There was no significant difference in RFS between the subgroups A1 and A2 (*p* = 0.560).

### 3.3. Univariate and Multivariate Analysis

In multivariate Cox regression analysis, after adjusting factors such as BMI, age, gender, and gross hematuria history, IVC regimen after a second TURBT (*p* = 0.012) and T stage after a second TURBT (*p* = 0.005) were both independent factors for predicting RFS in patients with NMIBC (Table 2).

## 4. Discussion

The five-year recurrence rates of NMIBC after TURBT is 50–70%. To reduce the likelihood of recurrence, both the AUA and EAU guidelines recommend that NMIBC patients receive a single IVC immediately after the initial TURBT. Patients with immediate- and high-risk for recurrence should receive an IVC induction phase for 4 to 6 weeks and maintenance phase IVC for 7 to 12 months [13,14,15,16].

There is a significant risk of a residual tumor after an initial TURBT for a Ta/T1 stage lesion [17]. A residual tumor after the first resection leads to a higher recurrence rate after TURBT. Approximately 40% of patients with high-grade Ta and more than half of those with T1 tumors have residual lesions after the initial resection [18,19]. The EAU guidelines recommend that a second TURBT should be performed within 2-6 weeks of the initial resection, and should include a resection of the primary tumor bed. A second TURBT can increase the RFS. Eroglu et al. established that patients with a second TURBT had significantly higher 5-year and 10-year RFS (59.4% and 54.8% vs. 36.3% and 26.8%, respectively, *p* < 0.001) rates than those without a second TURBT [9].

IVC after a second TURBT is considered an effective treatment option to prevent recurrence [20]. However, there is no consensus on the standard regimen for IVC after a second TURBT. Commonly used chemotherapeutic agents in clinical practice include epirubicin and pirarubicin. Numerous studies and guidelines have demonstrated that there are no significant differences between these drugs in preventing disease progression [21,22]. 

IVC only reduces the risk of recurrence; it is not effective in reducing the risk of progression in intermediate- or high-risk patients. The EAU guideline recommends BCG immunotherapy [23,24,25]. BCG therapy is more effective in reducing the risk of tumor progression and recurrence, by approximately 27% and 32%, respectively. Five meta-analyses have confirmed that BCG vaccine IVC after TURBT is superior to TURBT alone or TURBT and chemotherapy for preventing NMIBC recurrence. Two meta-analyses have demonstrated that BCG vaccine therapy delays and potentially lowers the risk of tumor progression [26,27,28,29,30]. However, there are several problems with BCG vaccine IVC in China. First, it is not covered by basic medical insurance and patients may be financially burdened. Second, BCG vaccine therapy causes more toxicity and post-treatment side effects than IVC [31,32]. Third, the BCG vaccine is not available in most hospitals in China, and patients must purchase it from designated pharmacies in major cities. The BCG vaccine is shipped under extremely stringent conditions, which increases the medical risks. Fourth, there has been a recent global shortage in BCG vaccine supply [33,34]. Therefore, in our medical center, most intermediate- and high-risk patients with NMIBC are still administered chemotherapeutic drugs such as epirubicin and pirarubicin. Therefore, the BCG vaccine was not included in the scope of this study.

Similar to the IVC protocol after the first TURBT, SI should be adopted immediately after the second TURBT, which clearly indicates that SI effectively improves patient prognosis and recurrence. Perlis et al. concluded that an SI administered in the first 24 hours after TURBT significantly prolongs the RFS by 38% [35]. Sylvester et al. described a similar decrease in the odds of recurrence (39%) in patients receiving an SI immediately post-TURBT [36]. 

A controversy still remains about the subsequent IVC maintenance therapy after the second TURBT. The interval between the first and second TURBT is usually 2-6 weeks. During this period IVC maintenance therapy is continued. It is inconclusive whether the original IVC regimen should be continued after the second TURBT or whether the IVC induction phase should be restarted. This is a very important issue. Postoperative IVC leads to an increased chance of complications such as bladder irritation symptoms and urinary tract infections [29]. In addition, long-term IVC treatment may be a burden for some patients, due to its cost, inconvenience, toxicity, and possible carcinogenicity [37]. Therefore, unnecessary IVC should be avoided.

Our study is the first to explore IVC protocols after a second TURBT. Our results suggest that if there is no residual tumor in the tissue specimen after the second TURBT, there is no statistical difference in RFS between the two treatment regimens. Thus, there is no need to restart the IVC induction phase after the second TURBT. In contrast, for patients with a residual tumor in the pathological specimen after the second TURBT, a new IVC induction phase significantly decreased the risk of recurrence. Therefore, we believe that clarifying the pathological findings after the second TURBT is an essential step in deciding the IVC regimen. Our experience suggests that SI should be performed immediately after the second TURBT. If the pathological finding of the second TURBT specimen is benign, the initial IVC regimen should be continued. If the pathology of the second TURBT specimen reveals a residual tumor, the IVC induction phase should be restarted.

The current study has several limitations. First, data were collected retrospectively, which may have caused a selection bias. Although we adjusted for likely confounders, some extent of unmeasured confounding may have remained. Secondly, the study was conducted at a single center with a limited sample size, which may have affected the results. Thirdly, we used epirubicin and birubicin, and the usage was not uniform. In addition, the interval between the two TURBTs differed. These factors may have affected the results. Future prospective studies with a larger sample are needed to validate our results.

## 5. Conclusions

Based on our results, if the pathological findings of the second TURBT specimen is benign, patients can continue their previous treatment regimen without restarting a new round. Unnecessary IVC can be avoided in these patients. In contrast, for patients with residual tumors in the second TURBT specimen, the need to restart the IVC induction phase should be emphasized to improve patient prognosis.

## Figures and Tables

**Table 1:** Patient characteristics.

Variable, *n* (%)	Level	Total (*n* = 137)	Patient with no Residual Tumor (*n* = 85)	Patient with Residual Tumor (*n* = 52)	*p*
IVC regimen after 2nd TURBT	New round of IVC	78(57)	52(61)	26(50.000)	0.2
	Re-IVC induction regimen	59(43)	33(39)	26(50.000)	
BMI	<25	74(54)	46(54)	28(54)	0.975
	≥25	63(46)	39(46)	24(46)	
Age	≤70	89(65)	54(64)	35(67)	0.653
	>70	48(35)	31(36)	17(33)	
Gender	Female	32(23)	19(22)	13(25.000)	0.722
	Male	105(77)	66(78)	39(75.000)	
Smoking history	No	24(18)	18(21)	6(12)	0.150
	Yes	113(82)	67(79)	46(88)	
Risk group	Intermediate-risk	99(72)	61(72)	38(73)	0.569
	High-risk	38(28)	24(28)	14(27)	
Gross hematuria	No	20(15)	12(14)	8(15)	0.839
	Yes	117(85)	73(86)	44(85)	
1st T stage/Pathology grade	Ta/LG	21(15)	13(15)	8(15)	0.998
	Ta/HG	36(26)	23(27)	13(25)	
	T1/LG	7(5)	4(4)	3(6)	
	T1/HG	73(53)	45(53)	28(54)	
1st Tumor number	Single	83(61)	56(66)	27(52)	0.105
	Multiple	54(39)	29(34)	25(48)	
1st Maximum tumor diameter	<3cm	80(58)	54(64)	26(50)	0.119
	≥3cm	57(42)	31(36)	26(50)	
2nd T stage/Pathology grade	Ta/LG	20(15)	13(15)	7(13)	0.827
	Ta/HG	37(27)	23(27)	14(27)	
	T1/LG	5(4)	4(4)	1(2)	
	T1/HG	75(55)	45(53)	30(58)	

TURBT = transurethral resection; IVC = Intravesical Chemotherapy.

**Table 2:** Univariate and multivariate analysis of factors influencing recurrence.

Variables	Univariate HR	95%CI	*p*	Multivariate HR	95% CI	*p*
IVC regimen after 2nd TURBT						
New round of IVC	1			1		
Re-IVC induction regimen	5.834	(1.303, 26.123)	0.021	7.687	(1.555, 37.994)	0.012
BMI						
<25	1					
≥25	2.341	(0.829, 6.610)	0.108			
Age						
≤70	1					
>70	0.568	(0.203, 1.591)	0.282			
Gender						
Female	1					
Male	1.464	(0.407, 5.273)	0.560			
Smoking history						
No	1					
Yes	0.472	(0.101, 2.205)	0.340			
Gross hematuria						
No	1					
Yes	6.958	(0.903, 53.627)	0.063			
1st T stage						
Ta	1					
T1	0.727	(0.248, 2.128)	0.561			
1st Pathology Grade						
Low	1					
High	1.122	(0.312, 4.035)	0.860			
1st Tumor number						
Single	1					
Multiple	3.101	(0.924, 10.411)	0.067			
1st Maximum tumor diameter						
<3 cm	1					
≥3 cm	1.407	(0.479, 4.134)	0.535			
2nd T stage						
Ta	1			1		
T1	7.281	(1.631, 32.496)	0.009	9.218	(1.928–44.085)	0.005
2nd Pathology Grade						
Low	1					
High	0.863	(0.192, 3.883)	0.848			

TURBT = transurethral resection; IVC = Intravesical Chemotherapy.

## Data Availability

The dataset analyzed in this study is available from the corresponding author upon reasonable request.
